# Late-Stage Fabry Disease With Advanced Cardiomyopathy and Conduction Disorders

**DOI:** 10.7759/cureus.34414

**Published:** 2023-01-30

**Authors:** Saim Mustafa, Christopher J Schimmoeller, Benjamin Fleming, Pranav Venkataraman

**Affiliations:** 1 Internal Medicine, Virginia Tech Carilion Clinic, Roanoke, USA; 2 Cardiology, Virginia Tech Carilion Clinic, Roanoke, USA

**Keywords:** alpha-galactosidase a, non-st segment elevation myocardial infarction (nstemi), late gadolinium enhancement, globotriaosylceramide, fabry's disease

## Abstract

Fabry disease, a well-known X-linked disorder, can present as an elusive late-stage disease in women with challenging limitations to management. Risk stratification of patient populations for genetic testing, early detection, and advances in affordable clinical treatment are on-going. We present a case to further demonstrate the need for continued research. Our case involved advanced complications, including worsening diastolic heart failure and conduction disorders ranging from supraventricular tachycardia to severe heart block. The patient received goal-directed medical therapy as tolerated for her heart failure and ultimately needed a dual-chamber pacemaker with a defibrillator.

## Introduction

Fabry’s disease (FD) is a deficiency of the alpha-galactosidase enzyme leading to a buildup of globotriaosylceramide (Gb3). It has an X-linked transmission and shows variable clinical presentations in females [1]. The classic findings for FD patients may present with severe neuropathic pain, telangiectasias/angiokeratomas, corneal opacities, and renal dysfunction. In adulthood patients could present with cardiac or cerebrovascular involvement, which is a major cause of mortality for these patients [1]. Given the variable clinical presentation in females due to random lyonization, it can require a high degree of suspicion to make the diagnosis; this can be a critical diagnosis [2]. Learning objectives: (1) To better understand the Fabry disease spectrum for heterozygous females. (2) To continue awareness and interest in research for Fabry disease.

## Case presentation

History of presentation

**Figure 1:**
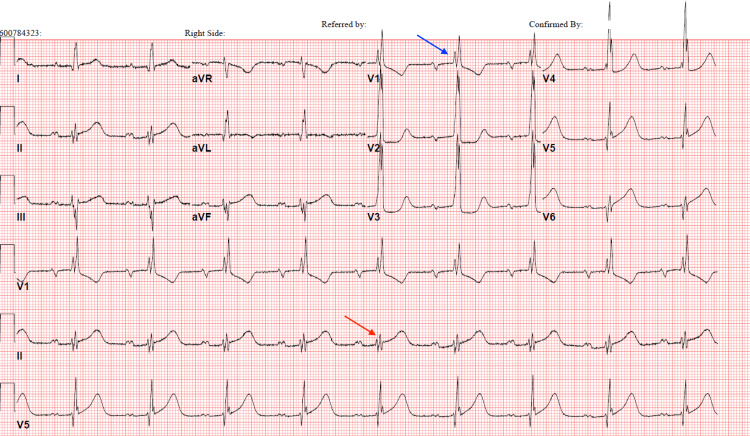
EKG on admission. Sinus bradycardia with first-degree AV block (red arrow), left atrial enlargement, and non-specific findings of intraventricular conduction block (blue arrow). PR, QRS, and QTc intervals are 228, 128, and 532 in milliseconds, respectively. EKG: electrocardiogram.

A 65-year-old African-American female was transferred for an escalation in care from a local community hospital for chest pain, non-sustained ventricular tachycardia (NSVT), and periods of heart block. Her chest pain was described as intermittent, pressure-like with radiation to her left arm, and on-going for the past 24 hours. She endorsed associated diaphoresis and dyspnea on exertion. The patient continued to endorse chest pressure, heightened anxiety, with on-going episodes of NSVT despite conservative therapy with anti-anginal medication. The physical exam was positive for bradycardia without appreciable murmurs, gallops, rubs as well as dysphagia without obvious oral mucosal or cavity findings. Otherwise, the patient was asymptomatic; there were no signs of heart failure (HF), no specific neurologic signs, and no classic skin findings of Fabry disease on a skin exam. She remained hemodynamically stable during admission. Electrocardiogram (EKG) was notable for sinus bradycardia, a historical right bundle branch block, a new first-degree atrioventricular block, and a new prolonged QTc (Figure 1).

Past medical history

The patient had a history of type II diabetes, hypertension, heart failure with a preserved ejection fraction of 70-75%, ischemic stroke 2020 with residual expressive dysphasia, chronic kidney disease stage III, and active tobacco abuse.

Differential diagnosis

This patient presented with significant cardiovascular risk factors for an acute coronary syndrome (ACS) event with non-ST elevation myocardial infarction high on the differential. Stress cardiomyopathy, intoxication, and vascular emergencies, including dissection, were also considered.

Investigations

High-sensitivity troponin resulted positive at 151 ng/L with a subsequent repeat at 155 ng/L. Lipid profile was within normal limit with low-density lipoprotein 64 mg/dL and high-density lipoprotein 79 mg/dL, hemoglobin A1C 5.5%, thyroid-stimulating hormone 2.9 µlU/ml. Chest x-ray was stable and negative for acute cardiopulmonary disease. The patient was started on an ACS heparin infusion with plans for cardiac catheterization. Angiography was notable for mild non-obstructive coronary artery disease (Videos 1, 2).

**Video 1:** Right coronary system angiography. Large right coronary artery without significant occlusion.

**Video 2:** Left coronary system angiography. Normal-sized left anterior descending artery and circumflex artery. Moderately tortuous vessels with mild luminal irregularities.

Management

**Figure 2:**
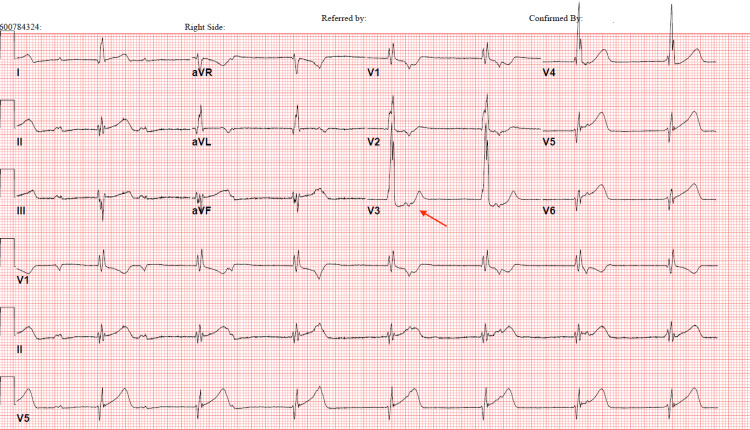
EKG after beta-blocker initiation. Sinus bradycardia with AV dissociation (red arrow) and wide QRS rhythm. Non-specific intraventricular conduction block continued. QRS and QTc intervals are 126 and 448 in milliseconds, respectively. EKG: electrocardiogram.

**Figure 3:**
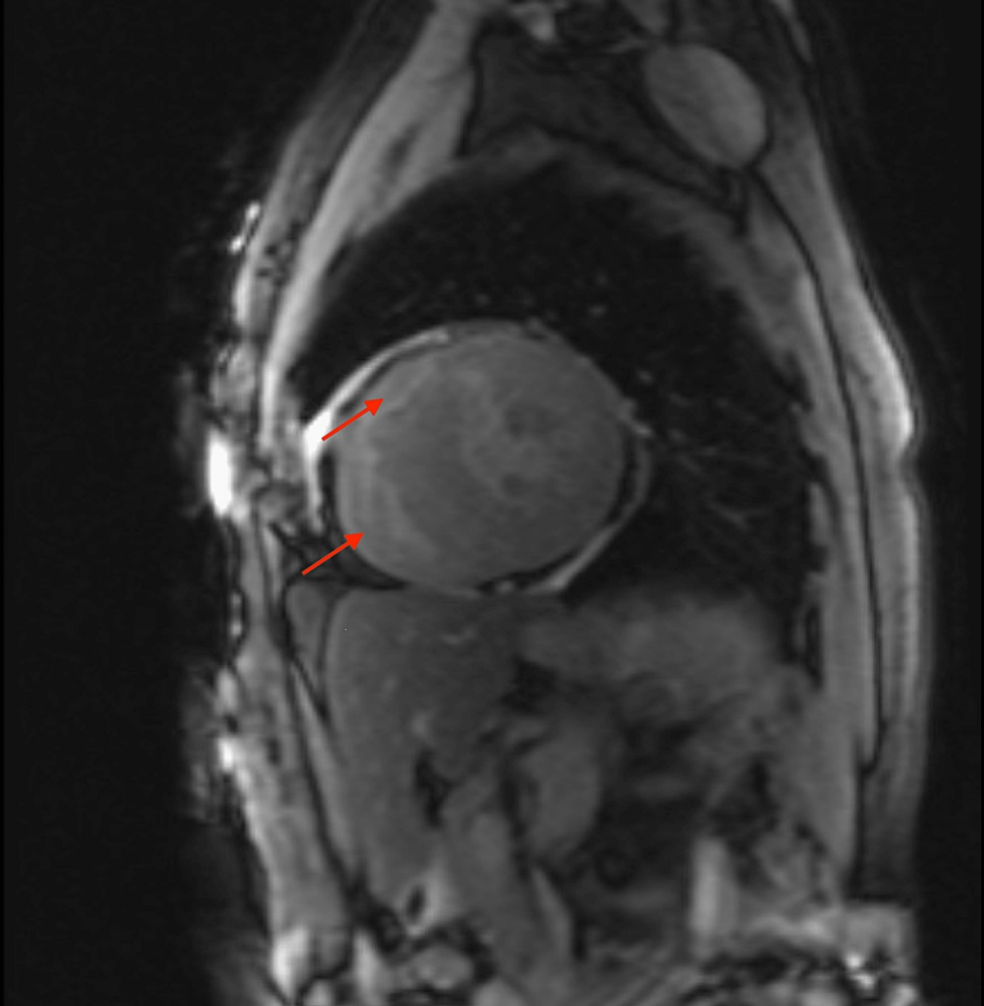
T1 Mapping on cMRI. Avid mid-myocardial LGE involving inferoseptal, anterior, and anterolateral segments (red arrows). LGE: late gadolinium enhancement, cMRI: cardiac magnetic resonance imaging.

Patient continued to have NSVT episodes. Oral metoprolol tartrate 25 mg was initiated with the subsequent development of a complete heart block (Figure 2). The transthoracic echocardiogram (TTE) was notable for a thickened left ventricle with an estimated ejection fraction of 65-70% (Videos 3, 4). Further investigation for infiltrative cardiomyopathy was pursued, including serum protein electrophoresis with immunofixation, serum quantitative kappa/lambda light, and random urine protein electrophoresis, which resulted negative. Given the concern for an amyloidosis impression on TTE, cardiac magnetic resonance imaging (cMRI) was ordered. The cMRI was markedly abnormal, and the impression was concerning for late-stage FD with patchy late gadolinium enhancement (LGE) in the left ventricle and extensive LGE involving the inferolateral base to apex region (Figure 3). The electrophysiology team placed a dual chamber pacemaker and implantable cardioverter defibrillator due to concern for worsening future bradyarrhythmia and sudden death. Patient was started on an angiotensin receptor blocker and restarted on beta-blockade with metoprolol succinate. Patient continued to progress well during her hospital course. Patient was discharged with plans for outpatient follow-up with the advanced heart failure team.

**Video 3:** Parasternal long axis. Severe concentric LVH. Significant increase in posterior left ventricular wall thickness. Preserved left ventricle cavity size. Aortic sclerosis without stenosis. LVH: left ventricular hypertrophy.

**Video 4:** Apical four-chamber. Hyperdynamic global left ventricular systolic function. Reduced average global longitudinal strain pattern of 8.87% with strain pattern consistent with apical sparing described as "cherry on top."

Follow-up

The patient followed up with the advanced heart failure clinic and electrophysiology providers. She remained stable with conservative medical management. On her subsequent device checks five months after discharge, she was found to have a high percentage of underlying atrial fibrillation, and she has since started anti-coagulation. She was later referred to nephrology for worsening kidney function with increasing proteinuria, persistent microscopic hematuria, and the development of metabolic acidosis requiring the initiation of bicarbonate therapy. Patient’s nephrologist ordered a renal ultrasound, which did not show any gross abnormalities. There are plans pending for a potential renal biopsy to further investigate her nephropathy and identify the involvement of FD.

## Discussion

FD was discovered in 1898, with the identification and characterization of a lysosomal origin for alpha-galactosidase enzyme deficiency later in the 1960s. Within the latter time frame, descriptions of cellular involvement and organ dysfunction were being described by the accumulation of "bodies" within cells, including endothelial, smooth muscle, and perivascular cells [3]. The first study to demonstrate a "high incidence" of cardiac involvement in heterozygous females was conducted in 1997. It showed a strong correlation with advancing age and the degree of LVH based on echocardiography. This study advocated that females should be included and considered as candidates for enzyme replacement therapy [4]. Myocardial dysfunction in males and females has also been depicted to differ. Female FD cardiomyopathy has an independent association with LVH compared to males. A 2011 study demonstrated that low systolic strain was always associated with LGE in either gender, but the severity differed among males and females in reflection of their LVH. The recommendations advocated by this study recommended initial cardiac staging in females based on LVH and fibrosis as opposed to the additional hallmark trait of myocardial dysfunction, described as systolic strain, previously characterized in male patients [5].

**Figure 4:**
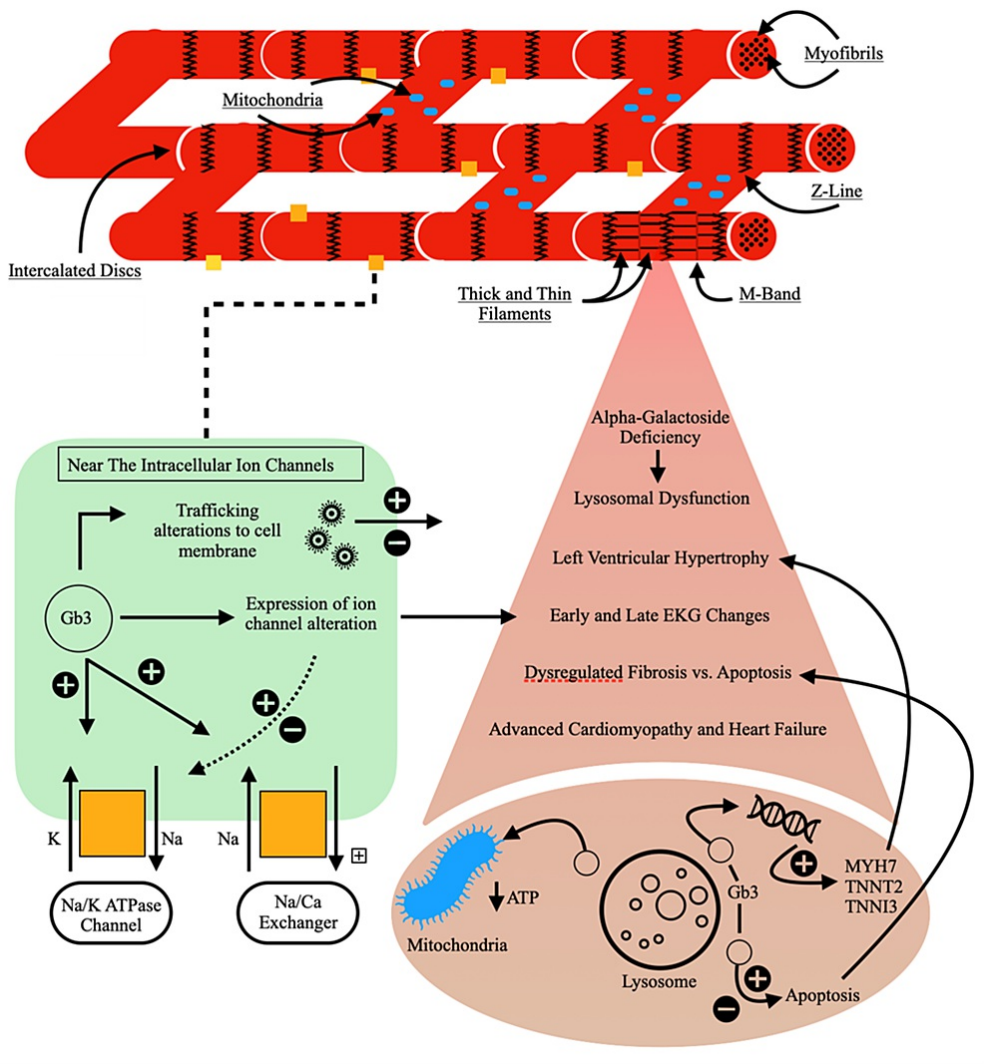
Cardiac myocyte dysregulation by Gb3. Intracellular processes affected by Gb3 include electrochemical pathways, organelle dysfunction, and alteration of gene expression. Gb3: globotriaosylceramide; MYH7: myosin heavy chain 7; TNNT2: cardiac troponin T; TNNI3: cardiac troponin I; ATP: adenosine triphosphate; EKG: electrocardiogram. Image original work of the authors.

The metabolic defect in FD involves a deficiency in alpha-galactosidase A (alpha-GAL A) [1,3]. This prevents the breakdown of the accumulating product, Gb3, into lactosylceramide and galactose. Of the 1000 and more identified variants of the galactosidase alpha gene, missense mutation variants are associated with late presentations of FD and have been identified as the predominant mutations in cardiac variants [1]. The phenomenon of random lyonization in females and their X-chromosomes results in phenotypic variations resulting in subsequent manifestations ranging from asymptomatic carriers to mild, moderate, and severe symptoms. These are typically classified by severity in their progression over time and the distribution of organ involvement. An earlier study demonstrated this phenomenon by evaluating the prevalence of lyonization. Using formally diagnosed female patients with FD, their X-chromosomes were analyzed with DNA methylation studies, and the prevalence of lyonization was determined. Afterward, they were able to corroborate their findings with subsequent imaging studies, including cMRI, and followed the progression of their cardiomyopathy and found a correlation with the degree of X-chromosome inactivation [2]. Gb3 accumulation alone does not explain the entirety of FD and organ dysfunction. On the contrary, its accumulation leads to a cascading effect of inflammatory processes, cellular proliferating and regulating processes, and unique intracellular dysfunctions that affect multi-organ systems differently [1]. These secondary processes in a cardiac myocyte are depicted in Figure 4. Fabry's disease continues to be discussed as a spectrum of disease in the heterozygous female, with its genetic mutations playing a role in disease severity.

Electrocardiographic manifestations of FD cardiomyopathy are non-specific but can be typified by PQ interval changes and repolarization abnormalities, which can precede LVH [1]. As LVH advances, left ventricular strain patterns can emerge with precordial T-wave inversions. Myocardial fibrosis, an important prognosticator of FD progression, may present as ST-T segment depression and T-wave inversion in inferolateral leads indicating posterolateral fibrosis [1]. Fibrosis can involve myocardial conduction tissue that could have led to the arrhythmias and conduction abnormalities seen in our patient (Figures 1, 2).

Concentric LVH and hypertrophic papillary muscles can be seen on echocardiography [6]. Importantly, right ventricular hypertrophy may also manifest in FD; however, the right ventricular function is classically preserved, which can be an important delineator between cardiac amyloidosis-a condition with right ventricular hypertrophy and reduced right ventricular function [6,7]. Abnormal left ventricular strain patterns have been well reported in the FD echocardiography literature, although it is without standardization: our patient’s reduced left ventricle-global longitudinal strain is common in FD but also in a variety of diseases with LVH. Loss of the base to apical gradient of circumferential strain may be a specific strain pattern seen in FD.

cMRI with LGE remains at the frontline of diagnostic imaging of FD with the classic pattern of mid-myocardial LGE of the basal and mid-inferolateral walls [6,7]. LGE signals can differentiate areas of fibrosis, with further T1 mapping being specific for Gb3 accumulation in the myocardium (Figure 3). Although seemingly non-specific, cardiac imaging can suggest further workup of FD cardiomyopathy in previously undiagnosed patients.

## Conclusions

Female FD lies on a spectrum that varies across patient populations and mutation severities. Awareness and recognition of symptoms are challenging, with the diagnosis being elusive until the late stages of the disease. Misdiagnoses and treatment delays have an obvious increase in mortality risk and pose a real threat to subsequent generations, previously misidentified as “carriers” only. We advocate for continued research in affordable treatment solutions and the implementation of early genetic marker testing for early diagnosis to prevent irreversible long-term effects in all populations, including undiagnosed heterozygous females.
